# CircGUCY2C regulates cofilin 1 by sponging miR-425-3p to promote the proliferation of porcine skeletal muscle satellite cells

**DOI:** 10.5194/aab-66-285-2023

**Published:** 2023-10-13

**Authors:** Kunlong Qi, Yaqing Dou, Chenlei Li, Yingke Liu, Chenglei Song, Xinjian Li, Kejun Wang, Ruimin Qiao, Xiuling Li, Feng Yang, Xuelei Han

**Affiliations:** 1 College of Animal Science and Technology, Henan Agricultural University, Zhengzhou 450046, China

## Abstract

Circular ribonucleic acids (or circRNAs) are an emerging class of endogenous noncoding RNAs that are involved in physiological and pathological processes. Increasing evidence suggests that circRNAs play an important regulatory role in skeletal muscle development and meat quality regulation. In this study, it was found that circGUCY2C exhibits a high expression level in the longissimus dorsi muscle. It shows resistance to RNase R and additionally promotes the mRNA expression of cyclin-dependent kinase 2 (*CDK2*) and proliferating cell nuclear antigen (*PCNA*). Specifically, it was observed that the overexpression of circGUCY2C could promote the transition of porcine skeletal muscle satellite cells into the S and G2 phases of the cell cycle and that it regulates the proliferation of porcine skeletal muscle satellite cells. In contrast, miR-425-3p plays the opposite role and has an inhibitory effect on the proliferation of porcine skeletal muscle satellite cells. MiR-425-3p has been described as a target of circGUCY2C; consequently, the depletion of miR-425-3p promoted the proliferation of porcine skeletal muscle satellite cells. *CFL1* (cofilin 1) is a target of miR-425-3p, and circGUCY2C upregulated *CFL1* expression by inhibiting miR-425-3p. Collectively, our research outcomes demonstrate that circGUCY2C significantly influences the proliferation of porcine skeletal muscle satellite cells by selectively targeting the miR-425-3p–*CFL1* axis, and our work partially clarified the role of circGUCY2C in porcine skeletal muscle satellite cells. Thus, the study provides new insight into the function of circGUCY2C and adds to the knowledge of the post-transcriptional regulation of pork quality.

## 1 Introduction

The types of circular ribonucleic acids (circRNAs) include exonic circRNA, intronic circRNA, exon–intron circRNA, and intergenic circRNA (Kristensen et al., 2019). CircRNAs have been initially recognized as aberrant splicing events in canonical and noncanonical RNA splicing (Cocquerelle et al., 1993). Although circRNA was discovered in 1976, after more than 40 years of study, the biological functions of circRNA still require elucidation and further research (Capel et al., 1993). Skeletal muscle satellite cells are a distinct cellular population that assumes a crucial function in the reparative and regenerative processes of skeletal muscle tissue. These cells are classified as stem cells within the skeletal muscle milieu, possessing the capacity for self-renewal and differentiation into myogenic cells. Upon encountering muscle stress induced by injury or exercise, skeletal muscle satellite cells undergo activation and differentiation, ultimately giving rise to skeletal muscle precursor cells that subsequently mature into functional muscle fibers. This process involves multiple regulatory factors and signaling pathways, including cytokines, growth factors, and transcription factors (Dumont et al., 2015). The proliferation and differentiation of satellite cells can modulate variables such as the number of myogenic cells, muscle fiber diameter, and endogenous muscle protein content, thereby influencing meat quality in livestock and poultry. A close genetic relationship exists between satellite cells and meat quality traits. Via differentiation into muscle fibers and by participating in muscle repair and regeneration, satellite cells and their regulatory mechanisms play a vital role in muscle development, repair, and the formation of meat quality characteristics (Brocks et al., 2000; Ryu and Kim, 2006). Thus, in-depth research into the genetic relationship between satellite cells and meat quality traits can contribute to improving the quality of livestock and poultry meat and meeting consumer demands, thereby playing a significant practical role in the development of the livestock and poultry industry. Furthermore, studies have revealed that circRNAs play a significant role in the proliferation and differentiation of skeletal muscle satellite cells. Specifically, circTCF4 has been shown to inhibit the proliferation and differentiation of goat skeletal muscle satellite cells (Zheng et al., 2022). Additionally, it has been found that circFNDC3AL upregulates *BCL9* (b-cell CLL/lymphoma 9) expression by binding to miR-204, thereby promoting the proliferation and differentiation of chicken skeletal muscle satellite cells (Wei et al., 2021).

In recent years, with the development of sequencing technology, an increasing number of noncoding RNAs (ncRNAs) have been discovered, and many circRNAs have been identified in mammalian cells using bioinformatics. CircRNAs are ncRNAs with a covalently closed-loop structure first discovered in viruses in the 1970s (Sanger et al., 1976). Many studies have found that circRNAs function as microRNA (miRNA) sponges (Pan et al., 2018), regulate alternative splicing (Ashwal-Fluss et al., 2014), regulate the transcription of parent genes (Zhang et al., 2013), act as a translation template for proteins (Pamudurti et al., 2017), and inhibit protein functions (Du et al., 2017). In addition, circRNAs are widely involved in the etiology of diseases including cancer, and life processes, such as body development (Li et al., 2017; Zhang et al., 2017; H. Liu et al., 2019). Due to the conservation of formation mechanisms related to circRNA, such as parent genes, splice mechanisms, and regulatory elements, circRNA exhibits greater conservation across different species. This conservation helps maintain the functionality and stability of circRNA, enabling it to play similar biological roles across various species. Unlike traditional linear RNA molecules that have 5' and 3' ends, circRNA molecules possess a covalently closed circular structure, rendering them unaffected by RNA exonucleases. This unique feature allows circRNA to demonstrate more stable expression and resistance to degradation by RNA exonucleases.

CircRNA is widely involved in the regulation of sarcomeres, cell proliferation, differentiation, and apoptosis as a competitive endogenous RNA (ceRNA) (Meng et al., 2023). Hsa_circ_001783 promotes the progression of breast cancer cells by sponging miR-200c-3p (Z. Liu et al., 2019). Research has revealed that hsa_circRNA_104348 is significantly upregulated in hepatocellular carcinoma tissues and cells that directly target miR-187-3p, thereby affecting the proliferation, migration, invasion, and apoptosis of hepatocellular carcinoma cells (Huang et al., 2020). Circ-PLXNA1 is expressed in duck adipocytes, leg muscle, and liver, and it attenuates the effect on *CTNNB1* (catenin beta 1) by binding miR-214, which in turn acts during adipocyte differentiation (Wang et al., 2020). CircINSR functions by sponging miR-15/16, thereby regulating the expression of target genes *FOXO1* (forkhead box O1) and *EPT1* (ethanolamine phosphotransferase 1), and modulating the adipogenic differentiation of preadipocyte (Shen et al., 2020). It has been found that bta_circ_03789_1 and bta_circ_05453_1 are potential miRNA sponges that can regulate *IGF1R* (insulin-like growth factor 1 receptor) and further influence the regulation of the longissimus-dorsi-related factors in bovine and that may have an important role in muscle growth and development (Yan et al., 2020). CircRNA_0000660–miR_693–*Igfbp1* (insulin like growth factor binding protein 1) can regulate hepatic lipid metabolism (Chen et al., 2020). CircFUT10 binding to let-7c promotes cell proliferation and inhibits cell differentiation by targeting *PPARGC1B* (peroxisome proliferator-activated receptor γ coactivator 1- β ) in bovine adipocytes (Jiang et al., 2020). In previous work, we also found that circSETBP1 can control the proliferation and differentiation of porcine intramuscular preadipocytes and 3T3-L1 cells by binding miR-149-5p and increasing the expression of target *CRTC1* (CREB-regulated transcription co-activator 1, where CREB refers to cAMP response element-binding protein) and *CRTC2* (CREB-regulated transcription co-activator 2) (Liu et al., 2023).

There has been considerable research showing that circRNAs can act as ceRNA and regulate the development of skeletal muscle satellite cells. CircPPP1R13B promotes the proliferation and differentiation of chicken skeletal muscle satellite cells and blocks the inhibitory effect of miR-9-5p on the proliferation and differentiation of chicken skeletal muscle satellite cells (Shen et al., 2021). CircTMTC1 inhibits chicken skeletal muscle satellite cell differentiation by sponging miR-128-3p (Shen et al., 2019). In addition, circRNAs can affect skeletal muscle development. CircSVIL can act as an miR-203 sponge and upregulate the levels of *c-JUN* and *MEF2C*, thus regulating myogenic cell proliferation and differentiation (Ouyang et al., 2018). CircEch1 may be a potential target for promoting bovine skeletal muscle development, inducing skeletal muscle regeneration and myogenic cell differentiation, and regulating beef quality (Huang et al., 2021).

In our previous study (Qi et al., 2022), circGUCY2C was identified as a differentially expressed circRNA in the longissimus dorsi muscle of Queshan Black and Large White pigs. By constructing a ceRNA network, we have discovered that the circGUCY2C–miR-425-3p–*CFL1* axis may potentially affect the meat quality in different pig breeds (Qi et al., 2022). However, the specific mechanism of action is still unclear and is the subject of this study. We confirm that circGUCY2C is a stable circular RNA; based on this information, we speculate that circGUCY2C affects skeletal muscle development in pigs. In this work, we investigate the ability of circGUCY2C to regulate the proliferation of porcine skeletal muscle satellite cells.

## 2 Materials and methods

### 2.1 Sample collection

For tissue expression pattern analysis, longissimus dorsi, adipose, liver, spleen, heart, lung, and kidney tissues were collected from Queshan Black ( n=3 ) and Large White ( n=3 ) pigs. Samples were rapidly frozen in liquid nitrogen and then stored at -80 ${}^{\circ }$ C.

### 2.2 Porcine skeletal muscle satellite cell isolation and culture

Porcine skeletal muscle satellite cells were isolated from the longissimus dorsi muscle of piglets aged less than 3 d. Briefly, tissue from the longissimus dorsi of the piglets was collected aseptically and washed several times in 75 % ethanol and PBS (phosphate-buffered saline) (Solarbio, P1020) containing 2 % penicillin–streptomycin. After removing the visible connective tissues in precooled PBS (Gibco, 10270-106), the muscle tissue was cut into 1 mm ${}^{3}$ pieces. The tissue was transferred into sterile centrifuge tubes and digested with 0.2 % collagenase type I for 90 min on a shaker with the digestion solution being blown every 15 min. The tissue was then digested with 0.25 % trypsin at 37 ${}^{\circ }$ C for 30 min with shaking and mixing every 10 min. The digestion was terminated with an equal volume of complete medium (10 % FBS, fetal bovine serum, + 1 % penicillin–streptomycin solution + DMEM, Dulbecco's modified Eagle's medium) (Solarbio, 12100) and passed through 70- and 200-mesh cell sieves in turn. The filtrate was centrifuged at 1000 rpm for 5 min, and cells were resuspended and inoculated into 60 mm cell culture dishes. The supernatant was aspirated into a new dish after incubation for 2 h at 37 ${}^{\circ }$ C in an incubator with 5 % CO ${}_{2}$ by volume, and the medium was changed every 2 d.

### 2.3 Identification of porcine skeletal muscle satellite cells by immunofluorescence staining

Paired box gene 7 (*Pax7*), a marker gene of skeletal muscle satellite cells, is expressed in both resting and proliferative phases; only when *Pax7* is normally expressed can it repair muscle damage. Therefore, in this study, the isolated and purified porcine skeletal muscle satellite cells were validated using this marker. Porcine skeletal muscle satellite cells were inoculated in 24-well cell culture plates; when the cell density reached about 80 %, the cells were rinsed with PBS, fixed with 4 % paraformaldehyde for 30 min, permeabilized with 0.1 % Triton X-100 for 30 min, and washed with distilled water. After blocked with 2 % goat serum for 1 h, samples were incubated with primary antibody (Anti-PAX7, 1:300 ) at 4 ${}^{\circ }$ C overnight. The primary antibody was removed and washed with distilled water, and a fluorescent secondary antibody (goat anti-mouse, 1:100 ) was added and incubated for 1 h. The secondary antibody was discarded and 250 µ L of DAPI (4',6-diamidino-2-phenylindole) staining solution was added dropwise and observed under a fluorescent microscope (Motic AE31E, China).

### 2.4 Vectors and transfection

According to the AUCGGGAAUGUCGUGUCCGCCC sequence of miR-425-3p, miRNA mimic and inhibitor were synthesized at GenePharma (GenePharma Co., Ltd., Shanghai, China). For the overexpression of circGUCY2C, we synthesized the entire linear sequence of circGUCY2C and inserted it into pLC5-ciR (Geneseed Biotechnology Co., Guangzhou, China), as per the manufacturer's instructions (p-circGUCY2C). The empty vector was set as control (pLC5-ciR). The wild-type (wt) and mutant-type (mut) linear fragments (circGUCY2C and 3′UTRs of *CFL1*) containing the miR-425-3p binding site were subcloned into psi-CHECK-2 luciferase expression vector. All constructed vectors were verified by sequencing. Cells were transfected by Lipofectamine 3000 reagent (Invitrogen, Carlsbad, CA, USA) according to the manufacturer's instructions.

### 2.5 Analyses of cell cycle and cell proliferation by flow cytometry

The analyses of cell cycle and cell proliferation in porcine skeletal muscle satellite cells were performed with flow cytometry. The porcine skeletal muscle satellite cells were plated into a six-well plate and transfected, each treatment had three independent replicates. The cells were washed twice using 1 × PBS and digested to obtain cell suspension for each well. Cells were pelleted, washed, and dissolved in PBS. The cell suspension was subsequently added dropwise into 70 % ice-cold ethanol and stored at 4 ${}^{\circ }$ C for 24 h. Thereafter, cells were centrifuged at 1000×g for 10 min, resuspended in 1 mL RNase solution, and incubated in water bath at 37 ${}^{\circ }$ C for 30 min. Propidium iodide was added to the cells and incubated in a water bath at 37 ${}^{\circ }$ C for 30 min in the dark. The fluorescence was quantified from single cells using a flow cytometer (BD FACSCelesta, USA).

### 2.6 RNA and DNA extraction and real-time quantitative polymerase chain reaction (qRT-PCR)

Total RNA was extracted from the samples with Trizol (catalog no. 15596026, Thermo Fisher Scientific, USA) according to the manufacturer's instructions, as previously described (Qi et al., 2022). RNA quality and concentration were assessed using a NanoDrop One/One ${}^{\text{C}}$ (Thermo Fisher Scientific, USA). A 1 % agarose gel was used to monitor RNA degradation and contamination. Total DNA was extracted using standard phenol–chloroform procedure. In brief, finely ground tissue samples were placed into 1.5 mL Eppendorf tubes, and lysis buffer was added followed by thorough mixing. The tubes were then subjected to a 60 ${}^{\circ }$ C water bath for 30–60 min and cooled to room temperature. An equal volume of chloroform was added, mixed, and centrifuged. The supernatant was transferred to a new Eppendorf tube containing an appropriate amount of isopropanol, mixed, and allowed to stand at -20 ${}^{\circ }$ C for 15–20 min. After centrifugation, the DNA pellet was obtained, washed with 75 % ethanol, air-dried, and dissolved in an appropriate amount of ddH ${}_{2}$ O. The dissolved DNA was stored at -20 ${}^{\circ }$ C for future use. The synthesis of complementary DNA (cDNA) was accomplished using a PrimeScript™RT reagent kit with gDNA eraser (perfect real time) (code no. RR047A, Takara, Beijing, China) to convert the total RNA to cDNA, with random hexamers (for mRNA and circRNA) and Bulge Loop RT primer of the Bulge Loop U6 qRT-PCR primer set for miRNAs (MQPS0000002-1-100, RiboBio, Guangzhou, China) according to the manufacturer's instructions. The primer sequences have been described previously (Qi et al., 2022). Next, qRT-PCR was performed using TB Green^®^ Premix Ex Taq ${}^{\mathrm{TM}}$ II (code no. RR820A, Takara, Beijing, China) on the CFX96 real-time PCR detection system (Thermo Fisher Scientific, USA). The expression of circRNA, miRNA, and the proliferation marker genes *CDK2* (cyclin-dependent kinase 2) and *PCNA* (proliferating cell nuclear antigen) were performed using qRT-PCR; *CDK2* and *PCNA* are both involved in cell cycle regulation and DNA replication. Meanwhile, *GAPDH* (glyceraldehyde-3-phosphate dehydrogenase, for mRNA and circRNA) and *U6* (for miRNA) were used as the normalization controls (Turabelidze et al., 2010; Khanna et al., 2019), and all reactions were carried out in triplicate. The biological and technical repeats were performed three times each. The 2 ${}^{-\Delta \Delta CT}$ method was used to calculate the relative expression levels of gene (Schmittgen and Livak, 2008).

### 2.7 Dual-luciferase assay

To detect binding between circGUCY2C, target genes, and miRNAs, a dual-luciferase reporter assay was performed. Briefly, the 293T cells (ATCC, catalog. no. CRL-3216) were co-transfected with miR-425-3p mimic + mut or wt vector or mimic negative control + mut or wt vector, respectively. After 48 h of transfection, the luciferase activity was measured using a dual-luciferase assay (catalog no. E1910; Promega Corporation) on a Multiskan FC microplate reader (Thermo Fisher Scientific, USA). Firefly luciferase activity in each group was normalized to *Renilla* luciferase activity.

### 2.8 Cell proliferation assay with a Cell Counting Kit-8 (CCK-8)

Cell proliferation was examined using a Cell Counting Kit-8 (Biosharp, Hefei, China). The cell suspension was diluted to $2\times 10^{3}$ mL and plated in 96-well plates. Cells were incubated for 48 h with four replicates. Then, 10 µ L of CCK-8 reagent was added to each well. A microplate photometer measured the absorbance of each well at 450 nm (Multiskan FC, Thermo Fisher Scientific, Shanghai, China).

### 2.9 RNA isolation of nuclear and cytoplasmic fractions

A PARIS Kit (Invitrogen, catalog. no. AM1921) was used to isolate the cytoplasm and nucleus RNA. Extracted RNA was used to perform qRT-PCR. The ratio of nuclear to cytoplasmic expression was then calculated: nuclear enrichment = 2 ${}^{\mathrm{Raw}\;\mathrm{Ct}\;\mathrm{Cytoplasm}-\mathrm{Raw}\;\mathrm{Ct}\;\mathrm{Nucleus}}$ ; cytoplasmic enrichment = 2 ${}^{\mathrm{Raw}\;\mathrm{Ct}\;\mathrm{Nucleus}-\mathrm{Raw}\;\mathrm{Ct}\;\;\mathrm{Cytoplasm}}$ . Ct values were not normalized to the differential abundance of the *U6* or *GAPDH* housekeeping genes between cellular compartments (Troskie et al., 2021).

### 2.10 RNase R assay

According to the manufacturer's instructions, an appropriate amount of total RNA was taken and divided equally into two parts: one half was used as a control and stored at -80 ${}^{\circ }$ C; the other half was treated with RNase R (Geneseed, Guangzhou, China) and incubated at 37 ${}^{\circ }$ C for 15–20 min. After treatment, the expression of circRNA and its parent gene in the cells was detected by qRT-PCR. For the quantitative calculation of circRNA in the digestion and control groups, *GAPDH* in the control group was used uniformly as a normalization control.

### 2.11 Statistical analysis

R (version 4.1.0) was used for statistical analysis (Chan, 2018), and data visualization was performed using ggplot2 (https://ggplot2.tidyverse.org/, last access: 3 August 2022). The data from this study are presented as the mean ± SEM (standard error of the mean). An independent t test and one-way ANOVA were used to compare the differences between different groups, and P<0.05 was regarded as statistically significant.

**Figure 1:**
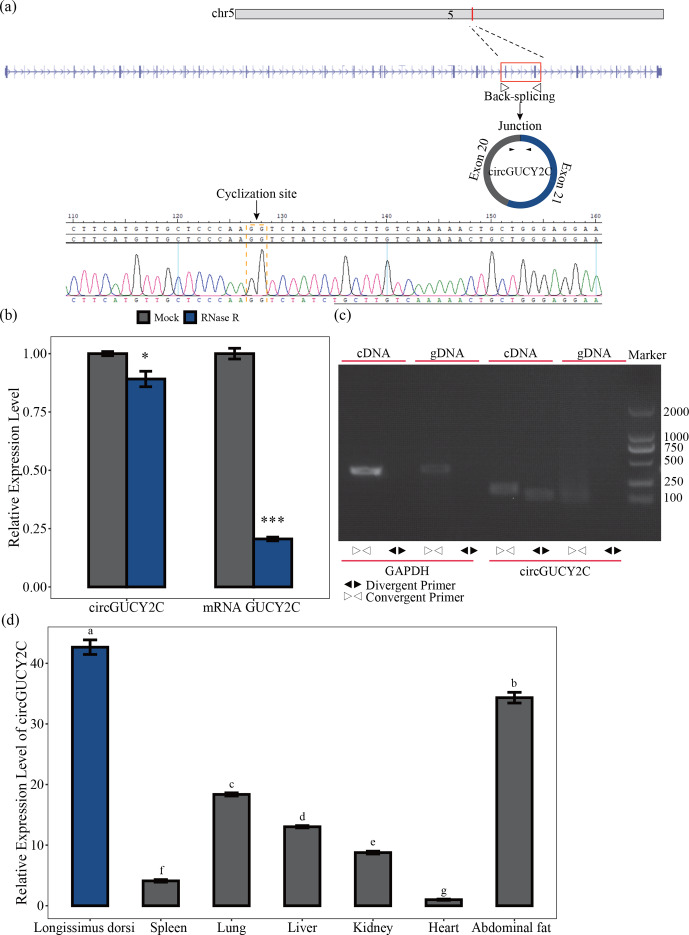
Identification and expression profile of circGUCY2C. Panel **(a)** provides a schematic representation of the formation of circGUCY2C and its genomic loci by cyclization of exon 20 and exon 21 of the *GUCY2C* gene. The back-splice junction sequence of circGUCY2C was verified by Sanger sequencing. Panel **(b)** shows the expression of circGUCY2C and linear *GUCY2C* under RNase R treatment ( n=3 ). One asterisk represents P<0.05 , two asterisks represent P<0.01 , and three asterisks represent P<0.001 . In panel **(c)**, RT-PCR was performed to detect the existence of circGUCY2C from cDNA and gDNA using the divergent and convergent primers. Panel **(d)** provides the expression of circGUCY2C in different tissues ( n=3 ). Different lowercase letters represent significant differences ( P<0.05 ).

## 3 Results

### 3.1 Identification and expression profile of circGUCY2C

A novel circRNA, named circGUCY2C, derived from the *GUCY2C* (guanylate cyclase 2C) mRNA on chromosome 5 and generated by the back-splicing of exons 20 and 21 was predicted by bioinformatics analysis based on previous research. We performed Sanger sequencing to confirm the back-splice junction site of circGUCY2C (Fig. 1a). CircGUCY2C was more resistant to RNase R treatment, and *GUCY2C* mRNA was significantly degraded by this treatment (Fig. 1b). Furthermore, PCR confirmed that different primers could amplify circGUCY2C from cDNA but not from gDNA (Fig. 1c). In addition, circGUCY2C expression was significantly higher in the longissimus dorsi compared with other tissues ( P<0.05 ; Fig. 1d). These results suggest that circGUCY2C might be a novel circRNA with a role in skeletal muscle in pigs.

### 3.2 CircGUCY2C promotes the proliferation of porcine skeletal muscle satellite cells

**Figure 2:**
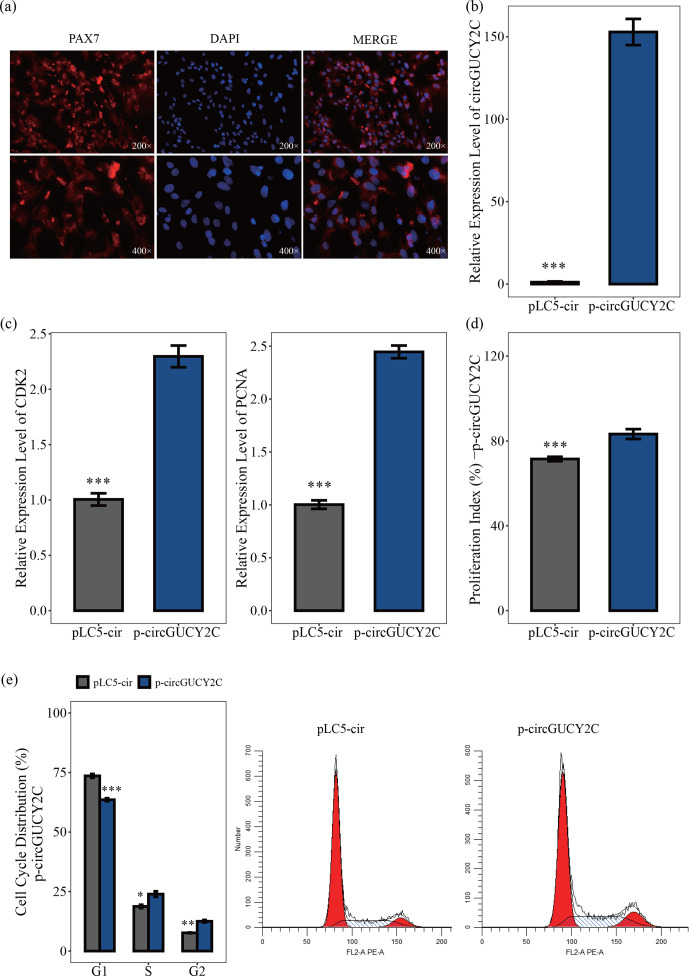
CircGUCY2C promotes the proliferation of porcine skeletal muscle satellite cells. Panel **(a)** provides an immunofluorescence identification of porcine skeletal muscle satellite cells. Panel **(b)** presents the expression level of circGUCY2C in porcine skeletal muscle satellite cells transfected with p-circGUCY2C and pLC5-ciR. This shows the overexpression efficiency of p-circGUCY2C compared with pLC5-ciR after transfection ( n=3 ). In panel **(c)**, the mRNA levels of proliferation-related genes were measured by qRT-PCR ( n=3 ). In panel **(d)**, the porcine skeletal muscle satellite cells' absorbance at 450 nm was detected by CCK-8 assay after the cells were transfected with p-circGUCY2C or overexpression plasmid (pLC5-ciR) ( n=4 ). Panel **(e)** presents a cell cycle analysis of porcine skeletal muscle satellite cells following circGUCY2C overexpression plasmid (pLC5-ciR) ( n=3 ). One asterisk represents P<0.05 , two asterisks represent P<0.01 , and three asterisks represent P<0.001 .

In this study, to investigate the effect of circGUCY2C on the proliferation of porcine skeletal muscle satellite cells, porcine skeletal muscle satellite cells were first isolated and purified. By using immunofluorescence on the obtained porcine skeletal muscle satellite cells, we then found that the positive rate of the marker PAX7 cells was high (Fig. 2a). The effect of circGUCY2C on porcine skeletal muscle satellite cell proliferation was investigated by qRT-PCR, CCK-8 assay, and flow cytometry. The overexpression vector p-circGUCY2C was constructed to manipulate the expression of circGUCY2C, and it was found that circGUCY2C was successfully upregulated in porcine skeletal muscle satellite cells ( P<0.01 ; Fig. 2b). Expression levels of proliferation-related genes including *CDK2* and *PCNA* were determined by qRT-PCR. Results showed that the overexpression of circGUCY2C upregulated the expression levels of these genes ( P<0.01 ; Fig. 2c). The CCK-8 assay showed that the proliferation index of porcine skeletal muscle satellite cells was significantly increased after the overexpression of circGUCY2C ( P<0.01 ; Fig. 2d). Furthermore, the cell cycle analysis revealed that the overexpression of circGUCY2C significantly reduced the proportion of cells in the G1 phase ( P<0.01 ) and promoted cell cycle progression to the S and G2 phases in porcine skeletal muscle satellite cells ( P<0.05 ; Fig. 2e). These suggest that circGUCY2C promoted the proliferation of porcine skeletal muscle satellite cells.

### 3.3 CircGUCY2C binds miR-425-3p

**Figure 3:**
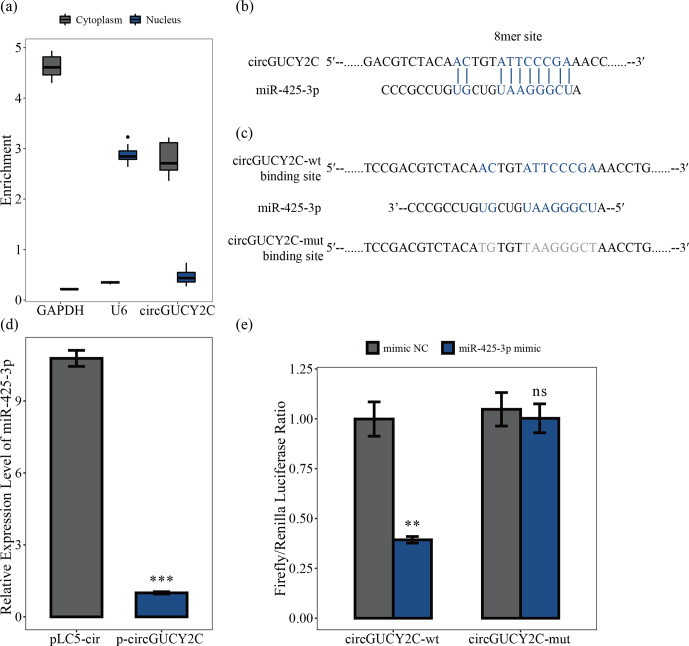
CircGUCY2C binds miR-425-3p. Panel **(a)** presents the circGUCY2C expression levels in the cytoplasm and nucleus of undifferentiated porcine skeletal muscle satellite cells ( n=4 ). Panels **(b)** and **(c)** show binding sites of miR-425-3p and circGUCY2C. In panel **(d)**, the expression levels of miR-425-3p were determined using qRT-PCR after porcine skeletal muscle satellite cells were transfected with circGUCY2C or overexpression plasmid (pLC5-ciR) ( n=3 ). In panel **(e)**, the firefly / *Renilla* luciferase ratio was detected after 293T cells were transfected with circGUCY2C-wt/circGUCY2C-mut or miR-425-3p mimic/mimic NC (negative control). One asterisk represents P<0.05 , two asterisks represent P<0.0 1, three asterisks represent P<0.001 , and ns represents not significant.

To determine the mechanism by which circGUCY2C regulates porcine skeletal muscle satellite cell proliferation, we analyzed the subcellular localization of circGUCY2C using a nucleocytoplasmic isolation assay, showing that the majority of circGUCY2C was localized in the cytoplasm (Fig. 3a). Therefore, the present study predicted the potential binding miRNAs of circGUCY2C, successfully predicted the miRNAs that may bind to circGUCY2C, and found that circGUCY2C has a stable binding site in the seed region of miR-425-3p (Fig. 3b). To further explore the interaction between circGUCY2C and miR-425-3p, we determined the expression of miR-425-3p using qRT-PCR after transfecting porcine skeletal muscle satellite cells with p-circGUCY2C, and results showed that the expression level of miR-425-3p was reduced by circGUCY2C overexpression ( P<0.01 ; Fig. 3d). Moreover, to clarify whether circGUCY2C directly binds to miR-425-3p, dual-luciferase reporter plasmids (psiCHECK-2 Vector) carrying wild-type or base-pair mutations in the binding site of circGUCY2C to miR-425-3p were constructed (Fig. 3c). Therefore, we validated the target relationship between circGUCY2C and miR-425-3p by detecting the ratio of firefly luciferase (F-Luc) to *Renilla* luciferase (R-Luc). Luciferase reporter assays showed that the firefly luciferase activity of circGUCY2C-wt was significantly reduced compared with that of circGUCY2C-mut after co-transfection with miR-425-3p into 293T cells (Fig. 3e). These findings suggest that circGUCY2C regulates miR-425-3p, indicating a negative regulatory effect of circGUCY2C on miR-425-3p.

### 3.4 MiR-425-3p represses the proliferation of porcine skeletal muscle satellite cells

**Figure 4:**
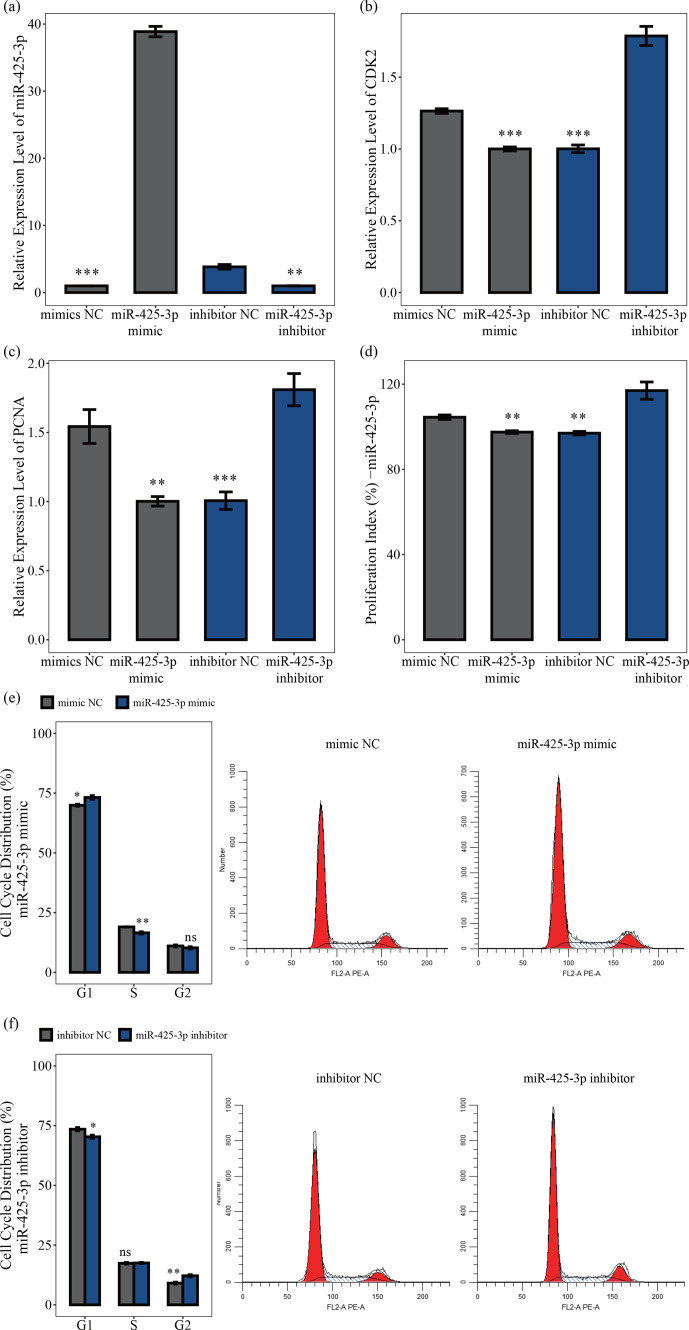
MiR-425-3p represses the proliferation of porcine skeletal muscle satellite cells. In panel **(a)**, the expression levels of miR-425-3p were determined using qRT-PCR after porcine skeletal muscle satellite cells were transfected with miR-425-3p mimic or inhibitor ( n=3 ). In panels **(b)** and **(c)**, the mRNA levels of proliferation-related genes were determined using qRT-PCR after porcine skeletal muscle satellite cells were transfected with miR-425-3p mimic or inhibitor ( n=3 ). In panel **(d)**, porcine skeletal muscle satellite cells' absorbance at 450 nm was detected by CCK-8 assay after the cells were transfected with miR-425-3p mimic or inhibitor ( n=4 ). Panels **(e)** and **(f)** display a cell cycle analysis of porcine skeletal muscle satellite cells following transfection with miR-425-3p mimic or inhibitor. One asterisk represents P<0.05 , two asterisks represent P<0.01 , three asterisks represent P<0.001 , and ns represents not significant.

MiR-425-3p mimic and inhibitor were used to explore the role of miR-425-3p in porcine skeletal muscle satellite cell proliferation. The qRT-PCR results showed that miR-425-3p expression could be significantly reduced by the miR-425-3p inhibitor but promoted by the miR-425-3p mimic ( P<0.01 ; Fig. 4a). To further understand the effect of miR-425-3p on porcine skeletal muscle satellite cell proliferation, we performed qRT-PCR, CCK-8 assay, and cell cycle analysis. The results showed that miR-425-3p mimic downregulated the mRNA expression levels of *CDK2* and *PCNA*, whereas miR-425-3p inhibitor upregulated the expression of these genes ( P<0.01 ; Fig. 4c, d). The CCK-8 analysis indicated that miR-425-3p mimic might inhibit porcine skeletal muscle satellite cell proliferation, whereas miR-425-3p inhibitor promoted the proliferation of porcine skeletal muscle satellite cells ( P<0.01 ; Fig. 4d). Furthermore, the cell cycle analysis revealed that miR-425-3p mimic effectively prevented porcine skeletal muscle satellite cells from entering the S and G2 phases, whereas miR-425-3p inhibitor promoted the division of porcine skeletal muscle satellite cells ( P<0.05 ; Fig. 4e, f). Thus, we confirmed that miR-425-3p inhibits the proliferation of porcine skeletal muscle satellite cells.

### 3.5 CircGUCY2C positively regulates *CFL1* expression by targeting miR-425-3p

**Figure 5:**
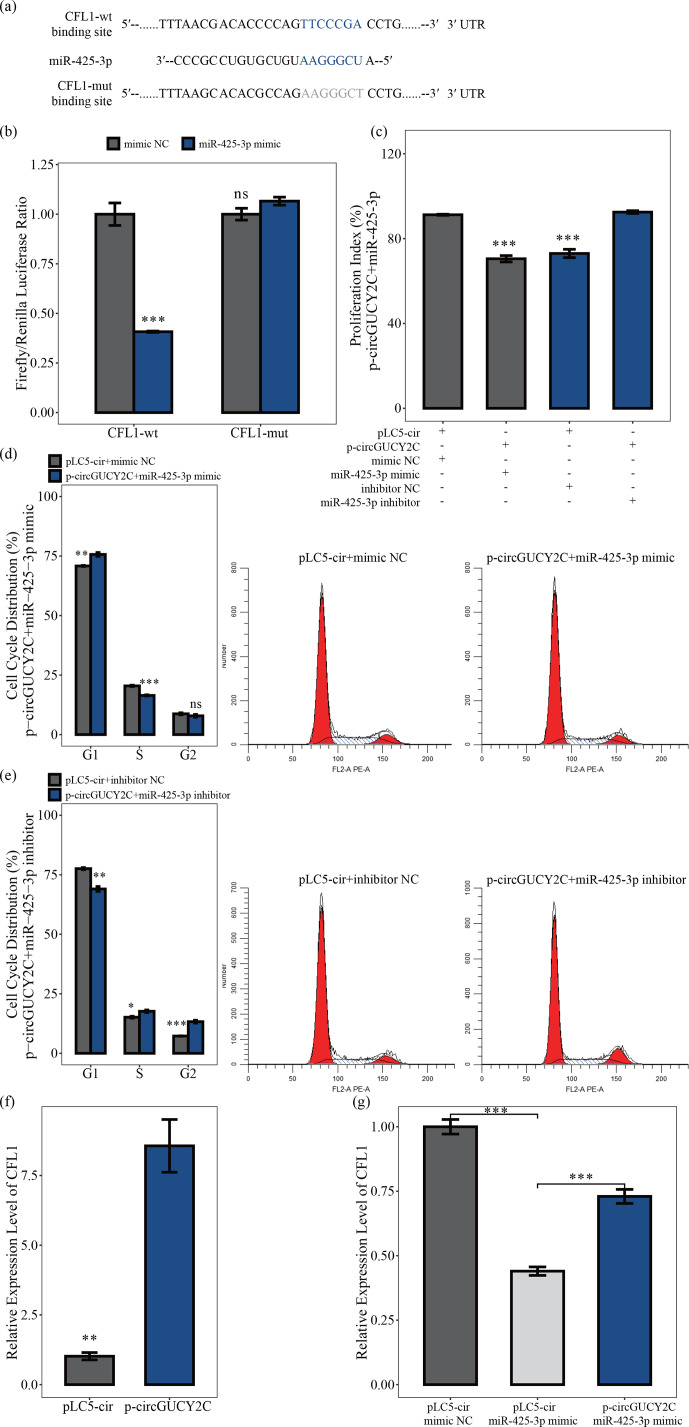
CircGUCY2C positively regulates *CFL1* expression by targeting miR-425-3p. In panel **(a)**, the target sites of miR-425-3p in *CFL1* mRNA 3' untranslated regions (3'UTRs) were analyzed. In panel **(b)**, the firefly / *Renilla* luciferase ratio was detected after 293T cells were transfected with *CFL1*-wt/*CFL1*-mut or miR-425-3p mimic/mimic NC. In panel **(c)**, the porcine skeletal muscle satellite cells' absorbance at 450 nm was detected by CCK-8 assay after transfection with circGUCY2C and miR-425-3p mimic or inhibitor ( n=4 ). Panels **(d)** and **(e)** present a cell cycle analysis of porcine skeletal muscle satellite cells following circGUCY2C and miR-425-3p mimic or inhibitor ( n=3 ). In panel **(f)**, the expression of *CFL1* was determined by qRT-PCR in porcine skeletal muscle satellite cells which were transfected with p-circGUCY2C or pLC5-ciR ( n=3 ). Panel **(g)** shows the co-transfection of circGUCY2C and miR-425-3p followed by qRT-PCR to detect the level of *CFL1* mRNA ( n=3 ). One asterisk represents P<0.05 , two asterisks represent P<0.01 , three asterisks represent P<0.001 , and ns represents not significant.

To determine the effects of the molecular mechanism of miR-425-3p on porcine skeletal muscle satellite cells, software was used to search for putative target genes of miR-425-3p, and *CFL1* was identified as a potential target gene of miR-425-3p (Fig. 5a). The luciferase reporter assay was performed to validate whether miR-425-3p binds to the 3′UTRs of *CFL1*. The luciferase activities of the *CFL1* 3′UTRs were significantly decreased after transfection with miR-425-3p mimic, but no change was observed in the mutated *CFL1* 3′UTRs, indicating that *CFL1* is a target of miR-425-3p ( P<0.05 ; Fig. 5b) In addition, to investigate whether circGUCY2C promoted the proliferation of porcine skeletal muscle satellite cells through binding miR-425-3p, a CCK-8 assay and flow cytometry were performed to detect cell proliferation in this study. After the co-transfection of circGUCY2C with miR-425-3p mimic, the proliferation index ( P<0.01 ; Fig. 5c) and the S and G2 phase of porcine skeletal muscle satellite cells were significantly decreased ( P<0.05 ; Fig. 5d), whereas the opposite results were obtained after co-transfection with miR-425-3p inhibitor (Fig. 5c, e). Furthermore, this study found that the mRNA levels of *CFL1* were upregulated after the overexpression of circGUCY2C ( P<0.01 ; Fig. 5f), and rescue experiments indicated that circGUCY2C could alleviate the miR-425-3p inhibitory effect ( P<0.01 ; Fig. 5g). These results suggest that circGUCY2C might reduce the inhibition of *CFL1* expression by the regulation of miR-425-3p, which in turn promotes the proliferation of porcine skeletal muscle satellite cells.

**Figure 6:**
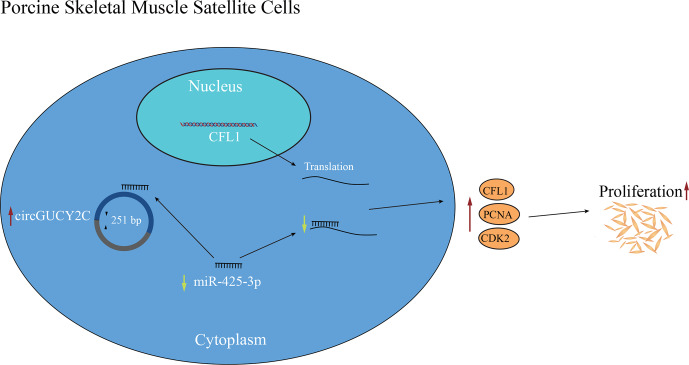
Schematic diagram of the regulatory mechanism of circGUCY2C–miR-425-3p–*CFL1* axis that regulates the proliferation of porcine skeletal muscle satellite cells.

## 4 Discussion

Skeletal muscle satellite cells are a type of cell present in skeletal muscle tissue, and they play a key role in muscle development and regeneration. During muscle growth and development in livestock and poultry, activated skeletal muscle satellite cells enter muscle tissue and proliferate and differentiate into myogenic cells; these cells subsequently fuse to form larger multinucleated muscle fibers, thereby promoting muscle growth and development. In addition, skeletal muscle satellite cells are also involved in the regeneration and repair process of muscle fibers. Thus, skeletal muscle satellite cells play a crucial role in the development of livestock meat quality (de Las Heras-Saldana et al., 2019; Bi and Kuang, 2012; Verdijk et al., 2007). In the present study, we found that circGUCY2C promoted the proliferation of porcine skeletal muscle satellite cells by upregulating the expression levels of proliferation marker genes *CDK2* and *PCNA*, increasing the proliferation index and the proportion of cells in the S phase. Therefore, circGUCY2C may be an important molecule in regulating the proliferation of porcine skeletal muscle satellite cells.

The regulatory mechanisms of protein-coding genes and noncoding RNAs on animal skeletal muscle cells and adipocytes have attracted considerable attention. Protein-coding genes have been extensively explored in terms of their regulatory functions, whereas the investigation of noncoding RNAs warrants further exploration. Although circRNAs lack protein-coding capacity, previous studies have indicated that they can still carry genetic information and exhibit important functionality. CircFAM188B encodes a novel protein, circFAM188B-103aa, which has a regulatory function in the proliferation and differentiation of chicken skeletal muscle satellite cells (Yin et al., 2020).

CircRNA is a class of circular noncoding RNA that is more stable due to its special structure. The phenotype of an individual organism is usually determined by genetic information genes, and the expression of some genes is regulated by circRNAs. In this study, we screened for differentially expressed circRNA (circGUCY2C) based on previous sequencing analysis and found that it exhibited higher resistance to RNase R compared with the parent gene *GUCY2C*. In addition, the cytoplasmic exon circRNA can act as an miRNA sponge to inhibit the function of its binding miRNAs and, thus, regulate gene expression (Wang et al., 2021). The present study also found that circGUCY2C is composed of two exons of the parent gene and is more expressed in the cytoplasm; therefore, this work also explored its role as an miR-425-3p sponge on the regulation of cell proliferation.

Most previous studies have focused on the ability of circRNA to regulate biological processes by acting as an endogenous RNA competing with miRNAs. In the cytoplasm, circRNA can act as a sponge for miRNA, interacting with miRNA to form a circRNA–miRNA regulatory network that has miRNA binding sites and can bind and inhibit the activity of miRNA; by this mechanism, circRNAs can bind miRNAs and thus regulate gene expression (Li et al., 2019). This study found the same mechanism around circGUCY2C. In this work, we found that circGUCY2C was localized in the cytoplasm and had a binding site for miR-425-3p, whose expression pattern is the opposite of that of miR-425-3p. Therefore, further experiments confirmed that circGUCY2C inhibited the expression and function of miR-425-3p through miRNA response elements and that circGUCY2C promoted the expression of proliferation-related genes, whereas miR-425-3p inhibited its expression. In addition, circGUCY2C overexpression promoted the expression level of *CFL1* mRNA.

Furthermore, this study suggested that circGUCY2C could regulate the expression of *CFL1* by targeting miR-425-3p. It was found that the overexpression of miR-425-3p inhibits the expression of *TGF*- β *1* (transforming growth factor beta 1), *p-smad2/smad2*, and *p-smad3/smad3* as well as the apoptosis of CVB3-HL-1 cells (Li et al., 2021). A total of 22 miRNAs with significant differences are found by miRNA microarray analysis, and miRNA-425-3p may be involved in lipid metabolism (Xu et al., 2019). Furthermore, results showed that *CFL1* was a target gene of miR-425-3p, and *CFL1* regulates adipocyte development (Kamal et al., 2013). In the present work, the results indicated that circGUCY2C was a positive regulator of porcine skeletal muscle satellite cell proliferation in pigs, suggesting that circGUCY2C has the potential to be an important marker affecting skeletal muscle development.

## 5 Conclusions

In the present study, a novel circRNA, named circGUCY2C, back-spliced by exons 20 and 21 of *GUCY2C*, was obtained, and it is highly expressed in porcine longissimus dorsi muscle. CircGUCY2C is a novel stable circular RNA found through experiments. CircGUCY2C promotes porcine skeletal muscle satellite cell proliferation by regulating the miR-425-3p and *CFL1* axis. In conclusion, we determined that circGUCY2C targets miR-425-3p to inhibit the negative effect of miR-425-3p on porcine skeletal muscle satellite cell proliferation (Fig. 6). Therefore, this work suggests that circGUCY2C may serve as a potential target factor for molecular breeding in pigs to improve skeletal muscle growth and that the circGUCY2C–miR-425-3p–*CFL1* regulatory axis may be a target for regulating skeletal muscle development. For livestock, this study expands our understanding of noncoding RNAs associated with porcine skeletal muscle satellite cell proliferation.

## Data Availability

The datasets analyzed and used in this study are available from the corresponding author upon reasonable request.
